# Physicians' perception about predictors of opioid abuse in patients with chronic non-cancer pain: a Delphi study

**DOI:** 10.3389/fpain.2023.1269018

**Published:** 2023-10-19

**Authors:** Santiago Galán, Rocío de la Vega, Rosa Esteve, Alicia E. López-Martínez, Mariano Fernández Baena, Carmen Ramírez-Maestre

**Affiliations:** ^1^ Instituto de Investigación Biomédica de Málaga (IBIMA Plataforma BIONAND), Málaga, Spain; ^2^ Red de Investigación en Cronicidad, Atención Primaria y Prevención y Promoción de la Salud (RICAPPS), Málaga, Spain; ^3^ Personalidad, Evaluación y tratamiento Psicológico, Facultad de Psicología y Logopedia, Andalucía Tech, Universidad de Málaga (Spain), Málaga, Spain; ^4^ Unidad del Dolor, Hospital Regional de Málaga, Málaga, Spain

**Keywords:** opioid, abuse risk, misuse, Delphi method, chronic pain

## Abstract

**Background:**

Opioids are being prescribed widely, and increasingly, for the treatment of chronic non-cancer pain (CNCP). However, several side effects are associated with mid- and long-term opioid use and, for certain patients, with the risk of problematic opioid use. The aim of this study is to know the perception of the physicians about which variables could be associated with increased risk of patients with CNCP developing a problem of abuse or misuse of the prescribed opioid medication.

**Methods:**

Twenty-nine physicians with experience in CNCP pain management and opioids prescription participated in a two-round Delphi study focused on the risk factors for opioid misuse and abuse.

**Results:**

The variables that reached consensus regarding their relationship with the increased risk of suffering a problem of opioid abuse or misuse were: (1) Experiencing pain on a daily basis, (2) previous use of high-dose opioids, (3) generalized anxiety, (4) hopelessness, (5) benzodiazepine intake, (6) use of opioids for reasons other than pain, (7) family problems, family instability or family breakdown, and (8) having access to several opioid prescribers. The only variable that reached consensus regarding it not being associated to a possible risk of abuse or misuse was having mild pain intensity (0–4 on a NRS-11).

**Conclusions:**

This study provides useful information that could help make decisions about the use of opioids for CNCP treatment and prevent future difficulties. Prospective studies testing the relationship of the variables that reached consensus with the risk of opioid misuse and abuse are warranted.

**Significance:**

This study shows the variables of CNCP that the professional must take into account in order to avoid possible problems when prescribing opioids.

## Introduction

Chronic non-cancer pain (CNCP) is defined as pain lasting or recurring for more than 3 months that is not due to a malignancy (1) and it has as a consequence a reduction in quality and quantity of life (2). It is a common problem, affecting more than 1 in 5 adults in America and resulting in elevated social costs (3). In Europe, the 1-month prevalence of moderate-to-severe CNCP reached 19% (4).

Despite multidisciplinary treatments being recommended as a first option of treatment (5, 6), opioids are being prescribed widely, and increasingly, for chronic pain management (7, 8). In Spain, the annual number of patients receiving at least one opioid prescription more than doubled, reaching to 722,838 in 2018 (9). The decision of conducting opioid prescription is not exclusively based on pain characteristics. Certain patient factors influence physicians’ decisions on using opioids, for example: being a woman (10), being older, reporting higher levels of pain intensity and depressive symptoms, reporting lower levels of pain-acceptance (10, 11), patients’ nonverbal communications of pain, distress, and suffering (12), patient´s trustworthiness, race and ethnicity, and the concern for risk of misuse (13), among others.

Unfortunately, a number of side effects, such as constipation, dizziness, and nausea, and serious side effects, such as addiction, are associated with the medium- and long-term use of opioids for CNCP (1, 14). The professional prescribing the opioids is also related to the risk of presenting side effects. For example, in comparation with other specialists, prescriptions from Primary Care providers have been associated with higher rates of opioid overdose deaths (15). Physicians’ knowledge of risk factors is also a variable to consider. Several studies have shown the relationship between the lack of knowledge of clinicians and stigma and also with a worse approach to long-term treatment (16, 17). Additionally, the use of opioids is controversial because of the risk of problematic opioid use (18) and the elevated risk of abuse and/or misuse (19). Misuse is defined as the use of opioid in a way that is different to the directed pattern of use, without considering the presence or absence of harm or adverse effects, and abuse is defined as the intentional use of the opioid for a nonmedical purpose, such as relaxation or altering one’s state of consciousness (20).

It has been shown that this risk is not the same for all patients; biopsychosocial patient factors such as: civil status, pain duration, mental health-related quality of life, and cigarette smoking, are associated with the risk of opioid abuse (21). Higher anxiety and depression levels have also shown to be significantly associated with increased opioid misuse (22) and also the presence of psychological disorders, such as post-traumatic stress disorder (PTSD) (5). However, there is a lack of research that provides a comprehensive overview of these variables.

A current systematic review have found a lack of consistent findings on the risk factors related to concurrent chronic pain and substance misuse (23). Given the lack of research, a Delphi methodology can provide evidence by generating consensus by a group of experts (24, 25). Therefore, we conducted a Delphi study to evaluate physicians’ perception regarding which variables could be associated with an increased risk of patients with CNCP developing a problem of abuse or misuse of the prescribed opioid medication.

## Material and method

### Study design

We conducted a Delphi study to address our aims. A Delphi method involves finding a group of experts in a specific topic and then asking them their opinions about a question of interest, in different and anonymous rounds. This method is frequently used to provide the most accurate answers to hard-to-answer questions (26, 27).

### Participants

In order to be eligible to participate as an expert, participants were required to: (1) attend patients with CNCP, (2) have expertise in opioid prescribing for chronic pain, and (3) speak Spanish. We used the snowball procedure to recruit experts into the Delphi panel (28). We first identified two experts who work in a Pain Clinic and we asked them to identify other experts who might be interested in participating. The group of experts should not be less than seven and the maximum is considered around 30 (29).

### Measures and procedures

This study was approved by the Institutional Ethics Review Board of the University of Málaga (CEUMA 66-2019-H) and the Regional Hospital Ethics Committee (CEI181021). It was conducted in accordance with the Declaration of Helsinki and Law 14/2007, of July 3, on Biomedical Research. The study information was sent via email or WhatsApp to potential participants. In this information, a link providing access to the informed consent form and the survey questions was added. The entire Delphi process occurred online via LimeSurvey. Participants did not receive any compensation for their time. Two rounds were needed to achieve consensus. Participants were the same in both rounds. Participating experts did not know the identity of the other members of the panel. Each round remained open for 1 month, and fortnightly reminder emails were sent. Two reminders per round were sent.

#### Round I

First, basic demographic and professional information was collected from the participants: age, sex, type of work center, position, years of experience and opioid prescription frequency. Using open-ended questions, panelists were then asked to identify all the variables that could be associated to the fact that a patient with CNCP could develop a problem of abuse or misuse of the prescribed opioid medication.

#### Round II

We presented all participants that had responded to Round I with the categories and items created in Round I. Participants were asked to rate the magnitude of the association of each item with an increased risk of suffering a problem of abuse or misuse on a scale of 1–10 (1 = Not at all associated, 10 = Extremely associated). The experts had the option to include new items if they considered that information was missing.

### Data analysis

#### Round I

We used means and ranges for continuous variables and numbers and percentages for categorical variables to summarize demographic and descriptive data from the study participants. We used the constant comparative analysis method (30) to qualitatively analyze and categorize data from the Round I open-ended question about the variables that could be associated to the fact that a CNCP could develop a problem of abuse or misuse of the prescribed opioid medication. Two of the authors (SG and RV) created the categories independently. Any disagreement was resolved by consensus. Those categories were then presented to the participants in Round II.

#### Round II

We recoded the ratings on the 1–10 scale as follows: responses ≤3 as “Not associated”, 4–7 as “Uncertain association”, and ≥8 as “Associated”.

We fist defined disagreement with the idea that a particular variable is related to increased risk of developing an opioid abuse or misuse problem if one-third or more participants responded in the “Not associated” range and one-third in the “Associated” range. Consensus was defined as the absence of disagreement. In the same line, at least 75% of the participants needed to respond with “Associated”, or “Not associated”, to determine that consensus was achieved for that item. This criterion is based on the median threshold used to define consensus in the Delphi method (31).

## Results

### Round I

Twenty-nine physicians based in Spain participated in the first Delphi round, 66% of these were men. The majority (83%), worked at a hospital and 66% were pain specialists. Their mean age was 38.2 years. Table 1 summarizes additional details regarding the demographic characteristics of the Round I participants.

**Table 1:** Characteristics of participants from round I (*n* = 29).

Age: mean (SD) and range	38.2 (12.3) 28–68
Sex: women (%)	34.5
Type of institution, *n* (%)	
Health Center	4 (13.8)
Hospital	24 (82.8)
Private Center	1 (3.4)
Position held	
Physician	10 (34.5)
Pain specialist	19 (65.5)
Clinical experience, mean years (SD)	15.4 (10.7)
Prescription opioid frequency, (%)	
Hardly ever	6.9
Rarely	10.3
Sometimes	44.8
Often	31.0
Always	6.9

Eleven categories were created from the answers obtained in the Round I open-ended question: (1) patient’s sociodemographic variables, (2) diagnoses and types of pain, (3) pain characteristics, 4) having other pathologies or diagnoses (different from the one that directly causes pain), (5) previous treatments that the patient had received for pain, (6) psychiatric history, (7) intellectual level, (8) psychological aspects that can influence the subjective perception of pain, (9) behaviors that the patient had had in the past, (10) possible alarm signs that the professional might observe in the patient’s behavior at the clinic visit, and (11) current circumstances surrounding the patient. The results of Round I are summarized in Table 2.

**Table 2:** Results from round I.

Categories	Item
Patient’s sociodemographic variables	
Sex	Woman
	Man
Age	Under 35 years old
	Between 35 and 65 years old
	Over 65 years old
Education Level	Low
	Medium
	High
Income Level	Low
	High
Marital Status	Single
	Separated
	Divorced
	Widow
Employment	Currently working
	On sick leave
	Unemployed
	Retired (due to age or illness)
Diagnoses and types of pain	
Primary Chronic Pain	Chronic generalized pain (such as fibromyalgia or non-specific musculoskeletal pain)
Secondary Chronic Pain	Chronic post-surgical or post-traumatic pain
	Chronic musculoskeletal pain
	Polyarticular pain
	Mechanical pain
	Chronic neuropathic pain
	Chronic nociceptive pain
	Mixed chronic pain
Lack of a clear Diagnosis	* *
Characteristics of pain	
Intensity (0–10)	Mild (0–4)
	Moderate (5–6)
	High (7–10)
Duration	3–6 months
	From 6 months to a year
	From one to 2 years
	More than two years
Frequency	Daily
	Several times a week
	Several times a month
	Less often than several times a month
Other pathologies or diagnoses (different from the one that directly causes the pain)	
	Degenerative diseases
	Obesity
	Sleep disturbances
	Immobility
	Inflammatory pathologies
	History of allergy to non-steroidal anti-inflammatory medications (NSAIDs)
Previous treatments that the patient has received for pain	
	Previous use of opioids for a long time
	Previous use of high-dose opioids
	Rapid pain reduction after opioid use
	Previous ineffective treatments other than
	opioids (e.g., failed back surgery, poor response to most NSAIDs, etc.)
	Have required rescue doses of medication
Psychiatric history	
	Generalized anxiety
	Hypochondria
	Depression
	Bipolar disorder
	Borderline disorder
	Histrionic personality
	Obsessive compulsive disorder
	Adaptive disorder
Intellectual level	
	Low
	Medium
	High
Psychological aspects that can influence the subjective perception of pain	
	Catastrophism/Concern for the future
	Hopelessness
	Unrealistic expectations
	Low pain tolerance
Others	Self-esteem issues
	Self-control problems
	Emotional dependence
	Not fearing continued opioid use
Behaviors that the patient has had in the past	
	Previous substance abuse (alcohol, tobacco, other drugs)
	Non-substance addictions (sex, video games, shopping)
	Non-scheduled use of analgesic medication
	Benzodiazepine intake
	Use of opioids for reasons other than pain (to sleep, to relax)
	Poor therapeutic adherence (not following the doctor’s recommendations)
	Failure to follow non-pharmacological recommendations (e.g., exercising, going to rehab)
Possible alarm signs that the professional may observe in the patient’s behavior at the visit	
	Showing high knowledge about opioids
	Insistence on the benefits of opioid therapy
	Physical appearance and self-care, hygiene, grooming
	Being very demanding (demanding quick results, rescue medication, reports, prescriptions)
	Rejecting proposals for other non-pharmacologic pain management alternatives
	Trying to demonstrate control over the pain situation and the management of medications
	Hiding data about their previous history
	High expressiveness of pain through gestures or complaints
	Altered behavior (restlessness, threatening, repetitive behavior, aggressiveness, irritability, anxiety, discomfort, impatient, emotional lability, anger)
	Inability to listen and/or being unreceptive
Current circumstances surrounding the patient	
	Economic problems
	Lack of family support
	Ongoing labor litigation or search for benefits (recognition of disability)
	Job problems (unemployment, precariousness or job dissatisfaction)
	Family problems, family instability or family breakdown
	Taking care of dependent people
	Social isolation, loneliness and/or lack of social support
	Substance abuse in the family
	Unhealthy habits and lifestyles (e.g., unbalanced diet, sedentary lifestyle, habitual consumption of tobacco and/or alcohol, etc.)
	Lack of resources to access non-pharmacological treatments (psychological/physiotherapy)
	Having access to several opioid prescribers (e.g., private and public insurance, pain unit)
	Malpractice of the professional who cared for them previously or who currently care for them (for example, not having followed the WHO pain ladder)
	Lack of patient follow-up in short times (i.e., not having frequent follow-up medical visits)
	Not understanding or speaking the local language correctly
	Being ineligible for surgery

Categories and subcategories of predictive variables included in the survey.

### Round II

Eighteen physicians (62%) of Round I participated in Round II. Despite being offered the option to add new information that had not been collected in the results of Round I, participants did not provide any new items. On the other hand, there was no disagreement between the participants, that is, no items were marked as unassociated by one-third or more participant responses AND one-third as associated by one-third participant responses.

The variables that reached consensus regarding their relationship with the increased risk of suffering a problem of opioid abuse or misuse were: (1) experiencing pain on a daily basis, (2) previous use of high-dose opioids, (3) generalized anxiety, (4) hopelessness, (5) benzodiazepine intake, (6) use of opioids for reasons other than pain, (7) family problems, family instability or family breakdown, and (8) having access to several opioid prescribers. The only variable that reached consensus regarding it not being associated to a possible risk of abuse or misuse was having mild pain intensity [0–4 on a Numerical Rating Scale (NRS-11)]. Finally, there were a number of variables that reached consensus regarding an uncertain association: (1) Medium education level, (2) type of pain: chronic nociceptive pain, (3) moderate pain Intensity, (4) pain duration from 6 months to a year, and (5) medium or (6) high intellectual level. The percentage item scores of each item are presented in Table 3.

**Table 3:** Results from round II.

Item	Associated	Not associated	Uncertain association
Pain frequency (Daily)	77.8		
Previous use of high-dose opioids	77.8		
Generalized anxiety	77.8		
Hopelessness	77.8		
Benzodiazepine intake	83.3		
Use of opioids for reasons			
other than pain	88.9		
Family problems, family instability			
or family breakdown	77.8		
Having access to several opioid			
prescribers	83.3		
Mild intensity (0–4)		77.8	
Education level (medium)			83.3
Chronic nociceptive pain			77.8
Moderate Intensity (5–6)			83.3
Duration (From 6 months to a year)			83.3
Intellectual level (medium)			77.8
Intellectual level (high)			83.3

## Discussion

In this study, we used a Delphi method to better understand the variables that could be associated with the risk that a patient with CNCP could develop a problem of abuse or misuse of the prescribed opioid medication.

Based on the patient’s history, there are a number of variables that the participating experts agreed that could be playing a role on opioid abuse or misuse risk. These variables were: having generalized anxiety, previous use of high-dose opioids and/or using of opioids for reasons other than pain (e.g., to sleep, to relax) and benzodiazepine intake. Those variables can be easily screened for by checking the patient’s medical record.

In line with the experts’ opinion, the association between the risk of opioid abuse or misuse and mental disorders has been well documented in the literature. For example, patients who report higher levels of psychological distress (anxiety and/or depression) and symptoms of a psychological disorder such as PTSD tend to be at higher risk of opioid misuse (5, 11, 22, 32, 33). Additionally, the history of use of high doses of opioids and benzodiazepines, or of opioids for other uses than those related to pain, are in line with the results obtained in other studies such as Skurtveit et al. with a sample of 17,074 participants. They suggest that earlier use of benzodiazepines may predict repeated use of opioids (34). In the same line, the results obtained by Cheatle et al. show that taking multiple doses of prescribed opioids together is related with misuse (35). But, not only in the past, a current opioid prescription has shown to increase the risk for opioid misuse (36, 37).

Based on the patient´s current circumstances, the variables that obtained the greatest consensus in our study were: a daily presentation of pain, having feelings of hopelessness, family problems, family instability or family breakdown, and having access to several opioid prescribers (e.g., private and public insurance, pain clinic, etc.).

Previous studies have evaluated the relationship between pain duration and the risk of abuse or misuse. In a longitudinal study, pain frequency appeared to be associated with an increased risk for opioid abuse or misuse /dependence, specifically, when the participant had been in pain for more than 16 days (6).

Our expert group agreed to find hopelessness related to the risk of opioid abuse or misuse. We have not found studies that link hopelessness with the risk of abuse or misuse (or to point to the opposite direction). However, there are studies that show the relationship between hopelessness and depression (38) and we have already shown the close relationship between depression and abuse or misuse, so we should consider assessing hopelessness as a potential risk factor. In addition, there are findings showing that hopelessness and substance abuse are related to PTSD symptoms severity (39). In fact, our results showed that the expert panel agreed to consider generalized anxiety and benzodiazepine intake as variables related with the increased risk of suffering a problem of opioid abuse or misuse. These variables have also been associated with PTSD (5).

Family problems, family instability or family breakdown have also been studied previously. A recent study with a sample of 8,103 students found that those who have family problems were significantly more likely to misuse opioids than students who did not report experiencing these relationship problems (40).

Finally, our expert group pointed out having access to several opioid prescribers as a risk variable. The study of Adewumi et al. is in line with these results and it also concludes that high doses of opioids at the start of treatment and low socioeconomic status are related to a high probability of having multiple prescribers (41).

Interestingly, our expert group agreed to find “mild pain intensity” as a non-risk variable. This finding is compatible with the work by Ives et al. and Cózar et al., that found no relationship between pain scores and opioid misuse (42).

The current study has several limitations that should be taken into consideration. Most of our results come from participants who work in the hospital setting (83%); No information on the level of training that experts had in addiction to opioid treatment was recorded. We do not know if these results would be the same if we had had more opinions from professionals from other fields such as primary healthcare centers or from other countries. This fact could also explain why some variables did not emerge, despite of that recent literature has pointed them out as key risk factors in predicting opioid abuse (e. g., the appearance of withdrawal symptoms and/or craving) (43–45).Therefore, future research could replicate these results considering the inclusion of this sample.

Future lines of research should: (1) evaluate the variables proposed by the experts and confirm whether there is real future risk of abuse or misuse in a prospective study and, (2) create and validate a questionnaire collecting the variables found in order to evaluate them systematically and easily.

Regarding the clinical practice, these findings may be useful for training physicians who prescribe or follow-up chronic pain patients using opioids to identify and minimize the risks associated with long-term opioid prescriptions. Furthermore, hospitals may benefit from screening their patients’ clinical records to look for the potential risk factors (e.g., prior opioid or benzodiazepine prescriptions) as well as considering conducting interviews with them to asses potentially negative current situations (e.g., family problems) or administering questionnaires to assess psychological variables (e.g., feelings of hopelessness) and, of course, assessing pain (intensity and frequency). As a result of this, additional monitoring could be put in place to follow-up on patients considered “at risk” in light of these variables.

Despite the dangers associated with the use of opioids in the treatment of CNCP, they are widely used. Clinicians have a very important role in this issue. This study provides useful information that could help them make decisions about the prescription of opioids for CNCP treatment and to monitor patients at risk of misuse and abuse, in order to prevent future difficulties.

## Data Availability

The raw data supporting the conclusions of this article will be made available by the authors, without undue reservation.
